# Luteolin-7-glucoside Promotes Human Epidermal Stem Cell Proliferation by Upregulating *β*-Catenin, c-Myc, and Cyclin Expression

**DOI:** 10.1155/2019/1575480

**Published:** 2019-06-02

**Authors:** Dapeng Wan, Yi Fu, Yingying Le, Ping Zhang, Jihui Ju, Benyuan Wang, Guangliang Zhang, Zhaodong Wang, Hao Su, Ling Wang, Ruixing Hou

**Affiliations:** ^1^Department of Hand Surgery, Ruihua Affiliated Hospital of Soochow University, Suzhou, China; ^2^Medical College of Yangzhou University, Yangzhou, China; ^3^Department of Human Anatomy, Histology and Embryology, School of Biology and Basic Medical Sciences, Soochow University, Suzhou, China; ^4^CAS Key Laboratory of Nutrition, Metabolism and Food Safety, Shanghai Institute of Nutrition and Health, Shanghai Institutes for Biological Sciences, University of Chinese Academy of Sciences, Chinese Academy of Sciences, Shanghai, China

## Abstract

Skin epidermal stem cells (EpSCs) play critical roles in skin homeostasis and the repair of skin injury. Luteolin-7-glucoside (L7G) has been reported to accelerate skin wound healing through its anti-inflammatory and antioxidative activity. But its effect on EpSCs is not clear. In the present study, we examined the effect of L7G on the proliferation of human EpSCs and explored the mechanisms involved. MTT assay showed that L7G promoted EpSC proliferation in a dose- and time-dependent manner. BrdU incorporation assay and Ki67 immunofluorescence staining confirmed the proproliferative effect of L7G on EpSCs. Cell cycle analysis showed that treatment of EpSCs with L7G decreased the cell number in the G1 phase and increased the cell number in the S phase. In addition, L7G significantly enhanced EpSC migration. Mechanistic studies showed that L7G significantly induced the expression of *β*-catenin and c-Myc, as well as cyclins D_1_, A_2_, and E_1_ which are critical for G1/S phase transition. L7G stimulated EpSC proliferation through *β*-catenin and c-Myc. We further examined the effect of L7G on EpSC proliferation in skin tissues by treatment of human skin explants with L7G and examined the number of EpSCs by immunohistochemical stain of EpSC markers *α*_6_ integrin and *β*_1_ integrin. We found that treatment of human skin tissue explants with L7G significantly increased the thickness of the epidermis and increased the numbers of *α*_6_ integrin-positive and *β*_1_ integrin-positive cells at the basal layer of the epidermis. Taken together, these results indicate that L7G promotes EpSC proliferation through upregulating *β*-catenin, c-Myc, and cyclin expression. L7G can be used to expand EpSCs for generating epidermal autografts and engineered skin equivalents.

## 1. Introduction

Epidermal stem cells (EpSCs) residing in the basal layer of the epidermis play a vital role in epidermal regeneration, skin homeostasis, and wound healing [1, 2]. When the skin is injured, the EpSCs around the wound are activated to proliferate, then the progeny migrate to the wound site and differentiate to regenerate the epidermis [3–5]. It has been reported that skin EpSCs self-renew via autocrine Wnt signaling [6] and Wnt/*β*-catenin signaling plays an important role in the proliferation of skin EpSCs [6, 7]. Other signalings also contribute to EpSC proliferation, such as integrin-MAP kinase- and Notch-related signaling pathways [8, 9].

EpSCs are becoming important sources for novel therapeutic approaches in the management of wounds. Transplantation of cultured keratinocytes which contain EpSCs, either in cell suspension or on various delivery systems, has been well established for the treatment of extensive, deep, and chronic wounds [10]. Furthermore, EpSCs are seed cells for skin tissue engineering to develop skin equivalents for skin grafting. Therefore, methods that permit obtaining a significant yield or expansion of EpSCs are required.

A large number of plants and plant extracts have been used in traditional medicines worldwide for the treatment of skin injury. Luteolin-7-glucoside (L7G) belongs to flavonoid glycosides. It is reported that L7G promotes skin wound healing by accelerating wound reepithelialization and increasing collagen deposition [11, 12]. The beneficial effect of L7G on wound healing is proposed to be mediated by its anti-inflammatory and antioxidative activity, as well as inhibition of the activity of hyaluronidase and collagenase [12]. In this study, we examined the effect of L7G on the proliferation of human EpSCs and explored the underlying mechanisms.

## 2. Materials and Methods

### 2.1. Reagents

Luteolin-7-glucoside was purchased from Nature Standard (Shanghai, China). XAV-939 and 10058-F4 were from Selleck (Houston, USA). Thiazolyl Blue Tetrazolium Bromide (MTT) was from Beyotime Biotechnology (Shanghai, China). Y-27632 was obtained from STEMCELL Technologies. The keratinocyte growth medium 2 (KGM2) was purchased from PromoCell (Heidelberg, Germany). Dispase II and collagen IV were from Sigma (Saint Louis, USA). The BrdU cell proliferation kit and antibodies against Ki67, CK19, *α*_6_ integrin, *β*_1_ integrin, PCNA, c-Myc, *β*-catenin, cyclin D_1_, and GAPDH were obtained from Abcam (London, UK). PI/RNase Staining Buffer, FITC-conjugated rat anti-human *α*_6_ integrin antibody, PE-conjugated mouse anti-human CD71 antibody, FITC-labeled rat IgG2a, and PE-labeled mouse IgG2a were from BD Biosciences (San Diego, USA). FITC-conjugated goat anti-mouse IgG antibody (BA1101) and Cy3-conjugated goat anti-rabbit IgG antibody (BA1032) were obtained from Boster (Wuhan, China). MaxVision™ HRP-Polymer anti-Mouse IHC Kit (KIT-5001) was provided by Fuzhou Maixin Biotech. Co. Ltd. (Fuzhou, China).

### 2.2. Isolation and Culture of Human EpSCs

Human skin was obtained from the extra fingers removed from male polydactyly patients (under 15 years old) during plastic surgery in Ruihua Affiliated Hospital of Soochow University, Suzhou, China. The protocol was approved by the Ethics Committee of the hospital. EpSCs were isolated from the skin tissue with dispase and trypsin as previously described [13, 14]. Briefly, subcutaneous fat of the skin tissues was removed, and the skin tissues were cut into 0.5-1 cm^2^pieces and digested with 0.25% dispase II overnight at 4°C. The epidermis was peeled off and digested with 0.25% trypsin for 10 min at 37°C. The keratinocyte cell suspension was filtered and centrifuged. The cell pellet was washed with PBS, resuspended in KGM2 supplemented with 0.125 ng/ml epidermal growth factor, 4 *μ*l/ml bovine pituitary extract, 0.06 mM CaCl_2_, and 100 IU/ml of streptomycin and penicillin, and seeded in 25 mm culture dishes coated with collagen IV. Cells were incubated for 10 min at 37°C in a humidified chamber with 5% CO_2_. Nonadherent cells were then immediately rinsed off. The adherent EpSCs were cultured with fresh medium supplemented with 10 *μ*M Y-27632 to promote EpSC proliferation and inhibit its differentiation [14, 15]. The second passage of EpSCs cultured in KGM2 without epidermal growth factor and Y-27632 was used in the following experiments.

### 2.3. Flow Cytometry

Human EpSCs were stained with FITC-conjugated rat anti-human *α*_6_ integrin antibody and PE-conjugated mouse anti-human CD71 antibody at 4°C for 1 h in the dark. FITC-labeled rat IgG2a and PE-labeled mouse IgG2a were used as isotype-matched negative controls. The cells were resuspended in PBS containing 2% FBS. FACS analyses were performed on a FACSCalibur (BD Pharmingen™).

### 2.4. Immunofluorescence Staining

The expression of biomarkers of EpSCs and Ki67 was examined by immunofluorescence staining. Briefly, EpSCs were fixed in 4% paraformaldehyde for 15 min, permeabilized with 0.1% Triton X-100, and incubated with normal serum from the same species as the secondary antibody for 30 min. Then, the cells were washed with PBS and incubated with the primary antibody overnight at 4°C. After washing, EpSCs were incubated with a fluorescence-conjugated secondary antibody for 1 h. The nuclei were stained with DAPI for 5 min. The fluorescence signal was detected with a fluorescence microscope (Olympus, Japan).

### 2.5. Cell Proliferation Assays and Cell Cycle Analysis

#### 2.5.1. MTT Assay

Human EpSCs seeded on 96-well plates were stimulated with different concentrations of L7G for different periods of time or stimulated with 1 *μ*M L7G combined with the *β*-catenin inhibitor (2 *μ*M XAV-939) or the c-Myc inhibitor (2 *μ*M 10058-F4) for 48 h. Twenty microliters of MTT at 5 mg/ml was added to each well, and the plate was incubated for 3 h at 37°C. The culture medium was removed, and 200 *μ*l of DMSO was added to each well to solubilize formazan crystals. The optical density (OD) was measured at 490 nm with the Multiskan™ Spectrum (Thermo Fisher Scientific Inc., Waltham, MA, USA). The experiments were performed in sextuplicate.

#### 2.5.2. BrdU Incorporation Assay

Human EpSCs seeded on 96-well plates were treated with or without 1 *μ*M L7G for 48 h. Cell proliferation was examined using the BrdU cell proliferation kit according to the manufacturer's instructions. The OD value was measured using the Multiskan™ Spectrum at 450 nm.

#### 2.5.3. Cell Cycle Analyses

Cell cycle phase distribution was analyzed by flow cytometry. Briefly, EpSCs treated with or without 1 *μ*M L7G for 24 h were trypsinized, washed with PBS, and then fixed in cold 80% ethanol. After the removal of ethanol by centrifugation, cells were stained with PI/RNase Staining Buffer (BD Biosciences, USA) for 20 min in the dark according to the manufacturer's protocol. The DNA content was analyzed by using a flow cytometer (Beckman Coulter).

### 2.6. Cell Migration Assay

The migration of human EpSCs was examined by in vitro wound closure assay. Human EpSCs (2 × 10^5^/well) were seeded on a 24-well culture plate. After overnight incubation, the confluent monolayer of cells was wounded with a 200 *μ*l pipette tip to create a uniform cell-free zone. Cell debris was removed by washing with PBS. Wounded monolayer was cultured in EGF free KGM2 with or without 1 *μ*M L7G. The cells were photographed under an inverted phase contrast microscope (Olympus, Japan) after 0, 12, and 24 h. The width of the cell-free zone was analyzed using the ImageJ software (NIH Image, Bethesda, Maryland, USA).

### 2.7. Reverse Transcriptase-Polymerase Chain Reaction (RT-PCR)

Total RNA was extracted from human EpSCs using the TRIzol reagent following the manufacturer's protocol (Invitrogen, Carlsbad, CA, USA). cDNA was reverse transcribed from RNA by using the oligo (dT) 18 primer and M-MLV reverse transcriptase (Takara, Dalian, China). PCR reaction was carried out with 35 cycles of 94°C for 30 seconds, 55°C for 30 seconds, and 72°C for 60 seconds. PCR products were separated by 1% agarose gel electrophoresis, visualized by ethidium bromide staining. The expression level of target genes was semiquantified with the ImageJ software. The sequences of PCR primers (5′-3′) are as follows: *β*-catenin: GCAGTTCGCCTTCACTATGGA (forward), ATCTTGTGGCTTGTCCTCAGAC (reverse); c-Myc: AGGAACTATGACCTCGACTACG (forward), AGTAGCTCGGTCATCATCTCCAG (reverse); cyclin D_1_: AACTACCTGGACCGCTTCCT (forward), CCACTTGAGCTTGTTCACCA (reverse); cyclin A_2_: TCCAAGAGGACCAGGAGAATATCA (forward), TCCTCATGGTAGTCTGGTACTTCA (reverse); cyclin E_1_: GTCCTGGCTGAATGTATACATGC (forward), CCCTATTTTGTTCAGACAACATGGC (reverse); and *β*-actin: CGTGGACATCCGCAAAGAC (forward), CTGCTGTCACCTTCACCGTTC (reverse).

### 2.8. Western Blotting

EpSCs were lysed in RIPA lysis buffer and centrifuged to remove the debris. The protein concentrations were measured using a Bradford protein assay kit (Beyotime, Beijing, China). Western blotting was performed following standard protocols. The primary antibodies against c-Myc, *β*-catenin, cyclin D_1_/E_1_/A_2_, and GAPDH were used. Target proteins were detected with SuperSignal West Pico Chemiluminescent Substrate (Thermo Fisher Scientific, Waltham, MA), quantified with the ImageJ software (NIH Image, Bethesda, Maryland, USA).

### 2.9. Skin Explant Culture

The normal skin explant culture was performed as described previously [16, 17]. Briefly, the human skin tissues were cultured in a 24-well transwell system with or without 1 *μ*M L7G in a 50/50 (*v*/*v*) mix of DMEM (1 g/l glucose) and Ham's F-12 medium (Gibco) supplemented with 10% FBS, 2 mM L-glutamine, and antibiotics. The epidermal side of the skin is kept at the air-liquid interface at 37°C under 5% CO_2_ in a humidified atmosphere, and the medium was changed every other day. After 2-5 days, the skin tissues were embedded in OCT compound (Sakura, USA) and frozen for histological and immunohistochemical assays.

### 2.10. Histology and Immunohistochemistry

Frozen human skin tissues were sectioned at 5 *μ*m using a cryostat (Leica) and stained with hematoxylin and eosin (HE). For immunohistochemical assay, the sections were fixed with acetone at 4°C for 10-20 min, incubated with 0.3% H_2_O_2_ for 15 minutes, washed with PBS, and blocked with goat serum for 30 min. Then, the sections were incubated with the primary antibody against *α*_6_ integrin, *β*_1_ integrin, or proliferating cell nuclear antigen (PCNA) at 4°C overnight, washed with PBS, and incubated with the secondary antibody from the MaxVison™ HRP-Polymer anti-Mouse IHC Kit for 15 min. After washing with PBS, the sections were incubated with the DAB-coloring agent (MAB, China) for 10 min in the dark, washed with water, and counterstained with hematoxylin. Sections were examined and photographed under a microscope. The positive signals of immunohistochemical stain were analyzed using the ImageJ software.

### 2.11. Statistical Analyses

All results are expressed as mean ± SD. Statistical differences between the test and control groups were analyzed with two-tailed Student's *t*-test. A *P* value of less than 0.05 was considered significantly different.

## 3. Results

### 3.1. Identification of EpSCs

**Figure 1:**
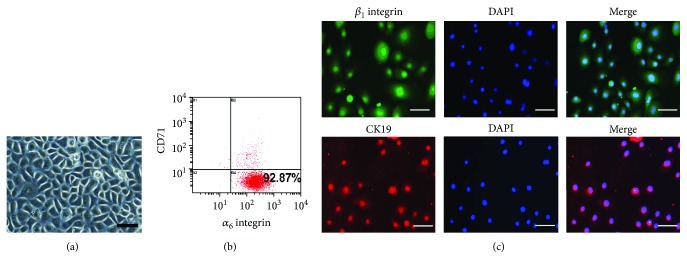
Characterization of human epidermal stem cells. (a) Morphology of cultured human epidermal stem cells (EpSCs) under a light microscope. Scale bar = 100 *μ*m. (b, c) The expression of epidermal stem cell biomarkers *α*_6_ integrin, CD71, *β*_1_ integrin, and CK19 in cultured EpSCs at the 2nd passage was examined by flow cytometry assay (b) and immunofluorescence staining (c), respectively. Scale bar = 200 *μ*m. Images are representative results of 3 independent experiments.

Human EpSCs were selected from epidermal keratinocyte suspension according to their rapid attachment to collagen type IV [18, 19]. After culturing in KGM2 with low concentrations of calcium (0.06 mM), these cells showed cobblestone-like morphology (Figure 1(a)). FACS analysis showed that these cells expressed high levels of *α*_6_ integrin and low levels of CD71, two well-recognized marks of EpSCs [19, 20]. The population of integrin *α*_6_^high^/CD71^low^ is higher than 90% (Figure 1(b)). We also examined the expression of *β*_1_ integrin and CK19, another two markers of EpSCs, by immunofluorescence staining, and found that more than 90% of the cells are *β*_1_ integrin and CKl9 positive (Figure 1(c)). These results demonstrated the successful isolation and culture of skin EpSCs.

### 3.2. L7G Promotes EpSC Proliferation and Migration

**Figure 2:**
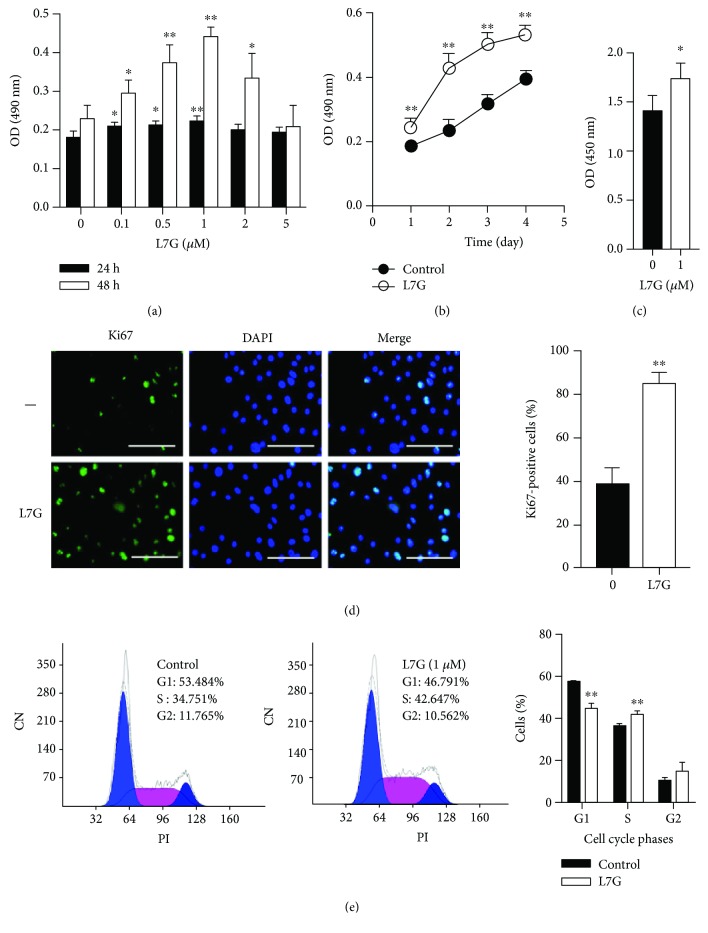
Luteolin-7-glucoside promotes human EpSC proliferation in vitro. Human EpSCs were treated with different concentrations of luteolin-7-glucoside (L7G) for 24 or 48 h (a) or treated with 1 *μ*M L7G for 1-4 d (b), 24 h (d), or 48 h (c); cell proliferation was examined by MTT assay (a, b), BrdU incorporation assay (c), or immunofluorescence staining of Ki67 (d). Cell cycle distribution of EpSCs treated with 1 *μ*M L7G for 24 h was detected by flow cytometry (e). Scale bar = 100 *μ*m. Data are shown as mean ± SD (*n* = 5). ^∗^*P* < 0.05 and ^∗∗^*P* < 0.01, compared with cells without L7G treatment for the same period of time.

To investigate the effect of L7G on EpSC proliferation, EpSCs were incubated with different concentrations of L7G and measured for cell proliferation by MTT assay after 24 and 48 h, respectively. We found that 0.1-1 *μ*M L7G promotes EpSC proliferation in a dose-dependent manner (Figure 2(a)). Stimulation of EpSCs with 1 *μ*M L7G for different periods of time showed that L7G enhanced cell proliferation in a time-dependent manner (Figure 2(b)). Treatment of EpSCs with L7G significantly increased the incorporation of BrdU (Figure 2(c)) and the number of Ki67-positive cells (Figure 2(d)). Furthermore, treatment of EpSCs with L7G resulted in a lower percentage of cells in the resting phase (G0/G1) and a higher percentage of cells in the proliferation phase (S phase) (Figure 2(e)). All together, these data demonstrate that L7G significantly promotes EpSC proliferation.

**Figure 3:**
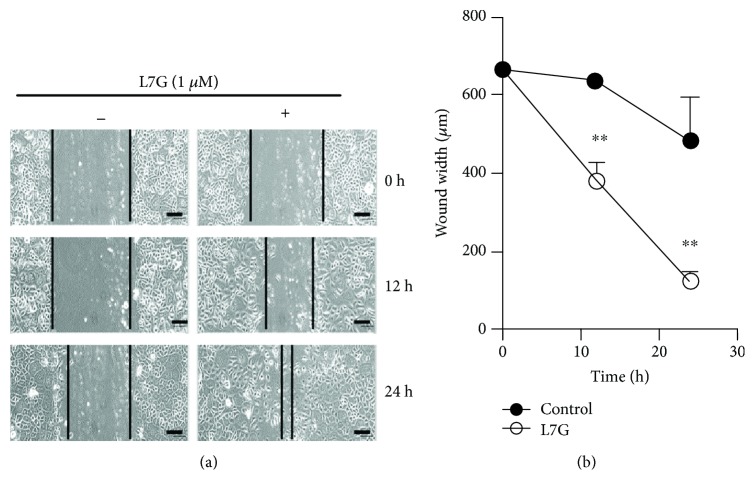
Luteolin-7-glucoside enhances human EpSC migration in vitro. A cell-free zone (wound) was made in confluent cultures of human EpSCs. The cells incubated with or without 1 *μ*M luteolin-7-glucoside (L7G) were photographed at different periods of time (a). Wound width (b) was measured with the ImageJ software. Data are shown as mean ± SD (*n* = 3). ^∗∗^*P* < 0.01 compared with untreated cells at corresponding time point. Images in (a) are representative results of three independent experiments. Scale bar = 100 *μ*m.

We further examined the effect of L7G on EpSC migration through the wound closure assay. Compared with untreated EpSCs, treatment of EpSCs with 1 *μ*M L7G for 12 or 24 h significantly accelerated cell migration and reduced the width of the scratched cell-free zone (Figure 3).

### 3.3. L7G Upregulates *β*-Catenin, c-Myc, and Cyclin Expression in EpSCs

**Figure 4:**
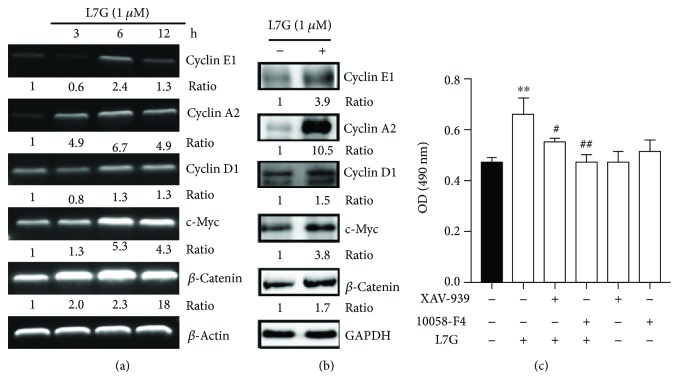
Luteolin-7-glucoside promotes EpSC proliferation through upregulating *β*-catenin, c-Myc, and cyclins. (a, b) Human EpSCs were treated with or without 1 *μ*M luteolin-7-glucoside (L7G) for different periods of time (a) or 24 h (b) and examined for the expression of *β*-catenin, c-Myc, and cyclins by RT-PCR (a) and Western blot (b), respectively. Images are representative results of 3 independent experiments. (c) Human EpSCs were treated with 1 *μ*M L7G combined with 2 *μ*M XAV-939 or 10058-F4 for 48 h. Data are shown as mean ± SD (*n* = 5). ^∗∗^*P* < 0.01, compared with untreated cells. ^#^*P* < 0.05 and ^##^*P* < 0.01, compared with cells treated with L7G alone.

It has been reported that *β*-catenin and c-Myc play critical roles in EpSC proliferation [6, 21]. We examined the effect of L7G on the expression of these genes in EpSCs. The results of RT-PCR showed that treatment of EpSCs with 1 *μ*M L7G significantly and time-dependently induced *β*-catenin and c-Myc expression (Figure 4(a)). Cyclin is a family of proteins which play an important role in regulating the cell cycle. We found that in addition to *β*-catenin and c-Myc, L7G also increased the expression of cyclins D_1_, A_2_, and E_1_ at mRNA level (Figure 4(b)). The upregulation of *β*-catenin, c-Myc, and cyclins by L7G at protein level was confirmed by Western blot (Figure 4(b)). We further found that treatment of EpSCs with the *β*-catenin inhibitor XAV-939 or the c-Myc inhibitor 10058-F4 significantly inhibited L7G-induced EpSC proliferation (Figure 4(c)). These results demonstrate that L7G promotes EpSC proliferation through upregulating the expression of *β*-catenin, c-Myc, and cyclins.

### 3.4. L7G Promotes EpSC Proliferation in the Human Skin Tissue

**Figure 5:**
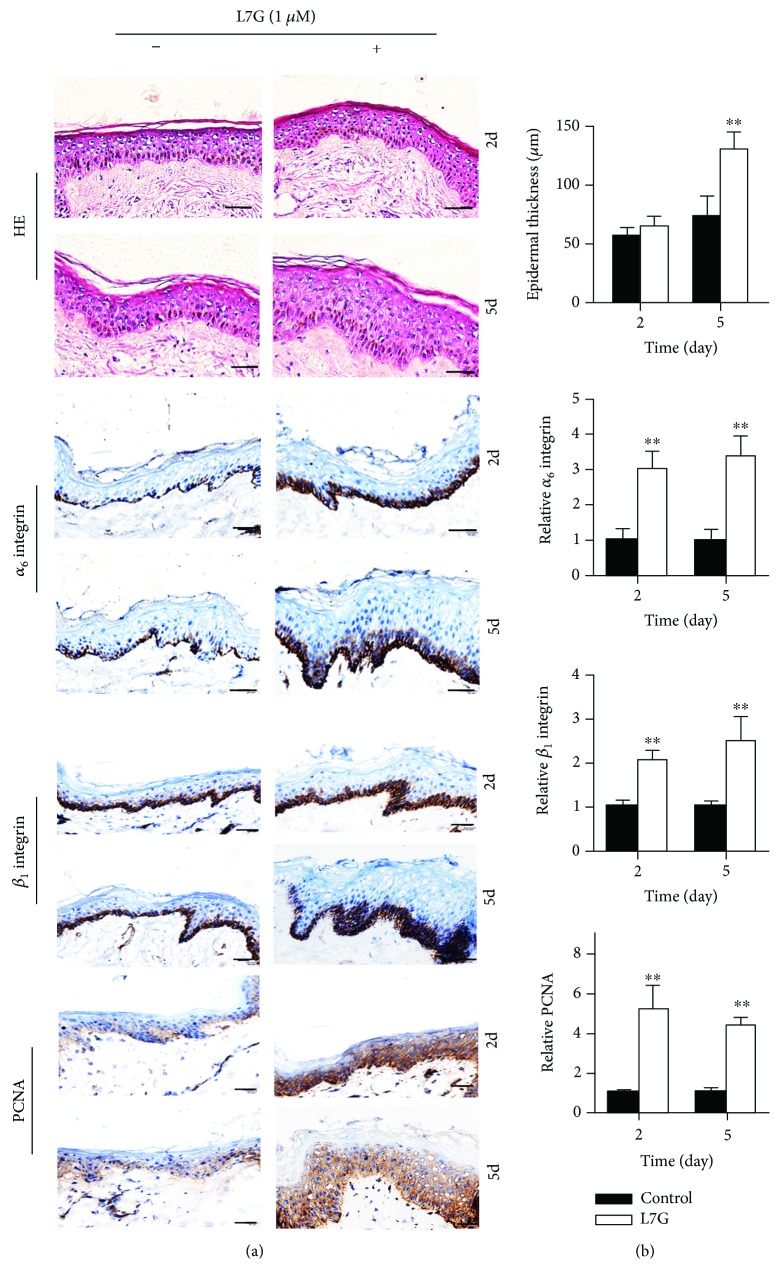
Luteolin-7-glucoside promotes EpSC proliferation in organotypic skin. Human skin explants cultured with or without 1 *μ*M luteolin-7-glucoside (L7G) for 2 and 5 days were examined for histology by HE staining and *α*_6_ integrin-positive, *β*_1_ integrin-positive, and proliferative cells by immunohistochemical staining. (a) Representative images. (b) Quantitative data of HE staining and immunohistochemical staining. *n* = 5; ^∗∗^*P* < 0.01 compared with untreated skin explants.

To investigate if L7G could promote EpSC proliferation in human skin, we cultured human skin tissue explants in medium with or without 1 *μ*M L7G for 2 and 5 days, respectively, then examined the histology by HE staining and the expression of marker genes of EpSCs and cell proliferation by immunohistochemistry. HE staining showed that treatment with L7G for 5 days significantly increased the thickness of the epidermis (Figure 5(a)). Immunohistochemical staining showed that in the untreated skin tissue, the cells expressing *α*_6_ integrin and *β*_1_ integrin, two markers of EpSC [22, 23], were located in the basal layer of the epidermis (Figure 5(a)). After treatment of the skin tissue with L7G for 2 and 5 days, the numbers of *α*_6_ integrin-positive and *β*_1_ integrin-positive cells and the staining intensity were significantly increased (Figures 5(a) and 5(b)). Treatment of skin tissues with L7G also increased the cells expressing PCNA, a nuclear marker of proliferating cells (Figures 5(a) and 5(b)). These results indicate that L7G promotes the proliferation of EpSCs in the skin epidermis.

## 4. Discussion

In the present study, we found that L7G promoted the proliferation of EpSCs in a concentration- and time-dependent manner and promoted EpSC migration in vitro. We further examined the effect of L7G on EpSCs in cultured human skin tissue explants. The immunohistochemistry results clearly showed that treatment with L7G significantly increased the staining of *α*_6_ integrin, *β*_1_ integrin, and PCNA in the basal layer of the skin epidermis, which is associated with the increased number of *α*_6_ integrin-, *β*_1_ integrin-, and PCNA-positive cells, as well as the thickness of the epidermis. These data strongly support that L7G could promote skin EpSC proliferation.

Our mechanistic study showed that L7G significantly induced the expression of *β*-catenin in EpSCs (Figure 4). The Wnt/*β*-catenin signaling has been reported to play important roles in the self-renewal and proliferation of skin EpSCs [5]. Deletion of the N-terminus of *β* catenin could avoid its binding and degradation by GSK-3*β*. Zhu and Watt reported that the introduction of the N-terminally truncated *β*-catenin into human EpSCs promoted EpSC proliferation and colony formation [24]. Jia et al. [25] reported that Wnt3a and *β*-catenin are expressed in the basal layer of human fetal skin and EpSCs. EpSCs also expressed c-Myc, cyclin D_1_, and cyclin A. Wnt3a stimulated the proliferation and inhibited the differentiation of human EpSCs, indicating that the Wnt3a/*β*-catenin pathway is important for EpSC proliferation. We found that the *β*-catenin inhibitor could block L7G-induced EpSC proliferation, indicating that L7G promotes EpSC proliferation through *β*-catenin. We further found that the expression of c-Myc and cyclin D_1_, two downstream molecules of *β*-catenin in cell proliferation, was also upregulated by L7G. c-Myc has been reported to be involved in skin EpSC proliferation [26]. Our study with the c-Myc inhibitor showed that L7G promotes EpSC proliferation through c-Myc. It has been reported that transgenic expression of cyclin D_1_ in the basal layer of mouse skin significantly induced epidermal cell proliferation [27]. In support by these results, our data indicate that L7G induces EpSC proliferation through Wnt/*β*-catenin-mediated c-Myc and cyclin D_1_ pathways.

We found that treatment of EpSCs with L7G decreased the cell number in the G1 phase and increased the cell number in the S phase, which verifies the proproliferative effect of L7G on EpSCs. Cyclin is a family of proteins which play an important role in regulating the cell cycle. Cyclin D_1_ drives G1/S phase transition. Cyclin A is required for G1/S phase transition, progression through the S phase, and also plays a role in G2/M phase transition. Cyclin E is essential for G/S transition [28, 29]. Our results showed that L7G upregulated the expression of cyclins D_1_, A_2_, and E_1_. These results indicate that L7G promotes EpSC proliferation by increasing G1/S phase transition. As cyclins A_2_ and E_1_ are not target genes of Wnt/*β*-catenin signaling, L7G may promote EpSC proliferation through upregulating cyclins mediated by Wnt/*β*-catenin/c-Myc-dependent and Wnt/*β*-catenin/c-Myc-independent pathways.

L7G promotes skin wound healing through its antioxidative and anti-inflammatory activity [12, 30, 31]. Although the relationship between oxidative stress and stem cells is not clear, it has been reported that the epidermal side population with stem cell-like characteristics exhibited less antioxidant proteins and produced lower levels of reactive oxygen species than more differentiated keratinocytes [32]. Choi et al. reported that vitamin C and plant extract with antioxidant activity increased the stemness and the proliferative potential of epidermal basal cells in cultured skin equivalents [33]. Therefore, it is of interest to examine if L7G could promote EpSC proliferation through the antioxidative pathway in the future.

## 5. Conclusion

L7G promotes skin EpSC proliferation through upregulating the expression of *β*-catenin, c-Myc, and cyclins D_1_, A_2_, and E_1_. Our results indicate that in addition to the antioxidative and anti-inflammatory activity, the proproliferative activity of L7G may also contribute to the acceleration of skin wound healing by L7G. L7G is a useful reagent for the expansion of EpSCs for the clinical translation of cell therapy and skin tissue engineering.

## Data Availability

The data used to support the findings of this study are available from the corresponding authors upon request.
